# Coagulation of β-conglycinin, glycinin and isoflavones induced by calcium chloride in soymilk

**DOI:** 10.1038/srep13018

**Published:** 2015-08-11

**Authors:** Yu-Hsuan Hsiao, Chia-Jung Yu, Wen-Tai Li, Jung-Feng Hsieh

**Affiliations:** 1 Department of Food Science, Fu Jen Catholic University, Taipei 242, Taiwan; 2 Ph.D. Program in Nutrition & Food Science, Fu Jen Catholic University, Taipei 242, Taiwan; 3 National Research Institute of Chinese Medicine, Ministry of Health and Welfare, Taipei 11221, Taiwan

## Abstract

The coagulation of β-conglycinin (7S), glycinin (11S) and isoflavones induced by calcium chloride was investigated. Approximately 92.6% of the soymilk proteins were coagulated into the soymilk pellet fraction (SPF) after the addition of 5 mM calcium chloride. SDS-PAGE and two-dimensional electrophoresis analysis indicated that most of the 7S (α’, α and β), 11S acidic (A1a, A1b, A2, A3 and A4) and 11S basic (B1a) proteins in the SSF were coagulated into the SPF after treatment with 5 mM calcium chloride. Isoflavones, including daidzein and genistein, were also coagulated into the SPF after the addition of 5 mM calcium chloride. The amounts of daidzein and genistein in the SSF decreased to 39.4 ± 1.6 and 11.8 ± 7.0%, respectively. HPLC analysis suggested that daidzein and genistein were bound with 7S and 11S proteins and then were coprecipitated into the SPF by 5 mM calcium chloride.

Soymilk is a colloidal solution containing 3.6% protein and is prepared by soaking and grinding soybeans in water, followed by filtering and heating. β-Conglycinin (7S) and glycinin (11S) are the two major soymilk proteins, representing approximately 40% and 30% of the total protein, respectively^1^. Recently, proteomics technologies, including sodium dodecyl sulfate polyacrylamide gel electrophoresis (SDS-PAGE) and two-dimensional polyacrylamide gel electrophoresis (2-DE) combined with protein identification by mass spectrometry (MS), have been used to study 7S and 11S proteins^2^. The 7S protein has a molecular weight (MW) of ~150 kDa and is a trimeric protein composed of three subunits (α’, α and β), which are assembled by hydrophobic forces and hydrogen bridges^3^. The other major storage protein, 11S, is a hexamer with a MW of 320–380 kDa. Five major subunits of 11S have been identified and classified into two groups, Group I (A1aB1b, A2B1a and A1bB2) and Group II (A3B4 and A5A4B3), based on the identities of their amino acid sequences. Each subunit of 11S is composed of an acidic (A) polypeptide with a MW of ~32 kDa and a basic (B) polypeptide with a MW of ~20 kDa, which are linked by an interchain disulfide bond^4^.

In addition to protein, soymilk is an excellent source of isoflavones. These isoflavones have received considerable attention due to their biological activity, such as lowering lipid and blood glucose levels, displaying antiatherosclerotic and antioxidation properties, and preventing hormone-dependent breast and prostate cancers^5^,^6^. For the analysis of isoflavones, high-performance liquid chromatography (HPLC) is the most widely used method for detecting and characterizing isoflavones^7^. Several isoflavones have been isolated from soymilk, and they can be found as non-conjugated β-glucosides (ex. daidzein, genistein and glycitein), conjugated malonyl β-glucosides (ex. malonyldaidzin, malonylgenistin and malonylglycitin) or β-glucosides (ex. daidzin, genistin and glycitin)^8^.

Soymilk can be transformed into soybean curd, also known as tofu. Tofu is a gel-like soybean food, formed by the addition of coagulants, resulting in the formation of a protein matrix^9^. Its preparation generally includes the coagulation of soymilk followed by molding and pressing. The coagulation of soymilk is the most important step in the tofu-making process^10^. Traditionally, calcium chloride is used as the coagulant in the industrial preparation of tofu. Coagulation occurs due to the cross-linking of protein molecules in the soymilk with the calcium chloride during incubation. Following the coagulation process, whey is removed for the preparation of a firm tofu^11^. The tofu coagulated with 0.4% calcium chloride was found to contain 1295.8 ± 2.4 μg isoflavones/g dry tofu^12^. These isoflavones included daidzin, glycitin, genistin, daidzein, glycitein and genistein. These results indicated that soymilk proteins and isoflavones were precipitated into tofu during the tofu-making process.

Although the soymilk proteins were coagulated by calcium chloride, it is unclear how the isoflavones were precipitated into the tofu during the production process. To investigate the effect of calcium chloride on the coagulation of 7S, 11S and isoflavones in soymilk, SDS-PAGE, 2-DE and HPLC were conducted. Therefore, the objective of this study was to investigate the calcium chloride-induced coagulation of 7S, 11S and isoflavones in soymilk.

## Results and Discussion

### Effects of calcium chloride on the coagulation of the soymilk proteins

**Figure 1:**
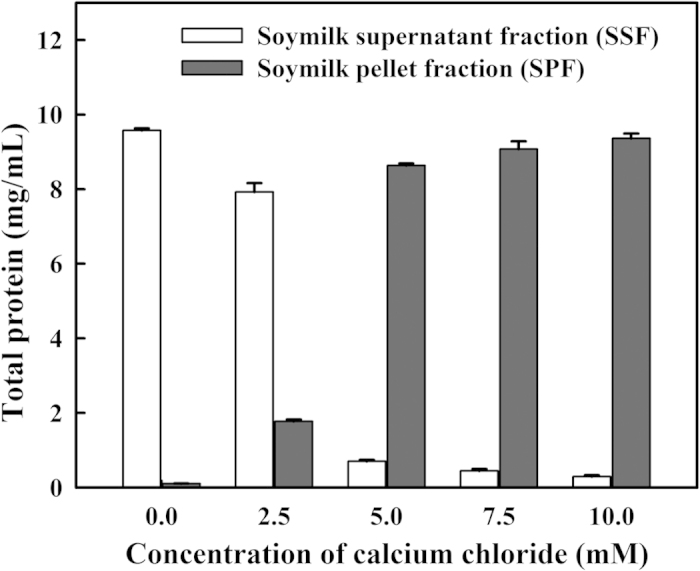
Changes in the total protein content of soymilk with different amounts of calcium chloride. SSF: soymilk supernatant fraction; SPF: soymilk pellet fraction. Vertical bars represent standard deviations.

Soymilk samples were incubated with varying concentrations of calcium chloride (0, 2.5, 5, 7.5 or 10 mM) for 1 h. The amount of total protein in the SSF and SPF was determined. As shown in Fig. 1, the total protein contents in the SSF and SPF, without calcium chloride treatments, were 9.57 ± 0.05 and 0.10 ± 0.04 mg/mL, respectively. This indicates that protein coagulation did not occur in the soymilk samples in the absence of calcium chloride. Following the 2.5 mM calcium chloride treatment, the total protein in the SSF decreased from 9.57 ± 0.05 to 7.92 ± 0.23 mg/mL. The total protein in the SSF decreased to 0.70 ± 0.03 mg/mL after the addition of 5 mM calcium chloride. Approximately 92.6% of the soymilk proteins were coagulated by the addition of 5 mM calcium chloride, and the total protein in the SPF significantly increased from 0.10 ± 0.04 mg/mL (without calcium chloride) to 8.63 ± 0.05 mg/mL (*P* < 0.05). The concentration of calcium in soymilk is very low (<0.02 mM) and that adding 25 mM calcium chloride to soymilk can precipitate soymilk proteins^13^. Ono^14^ reported that the coagulation of soymilk occurs due to the combination of calcium ions and soymilk proteins. The protein molecules bound by calcium ions have been described as being located on the side-chain carboxyl groups of aspartic and glutamic acid residues and the side-chain imidazole group of histidine residues. Ju and Kilara^15^ reported that Ca^2+^-induced protein aggregation is thought to arise from three effects: 1) electrostatic shielding, 2) ion-specific hydrophobic interaction, and 3) crosslinking of adjacent anionic molecules through the formation of protein-Ca^2+^-protein bridges. The binding of Ca^2+^ to soy proteins is an endothermic process (*ΔH* > 0) based on binding titration curves of soy protein dispersions with calcium chloride^16^.

### Analysis of the effects of calcium chloride on 7S and 11S proteins using SDS-PAGE

**Figure 2:**
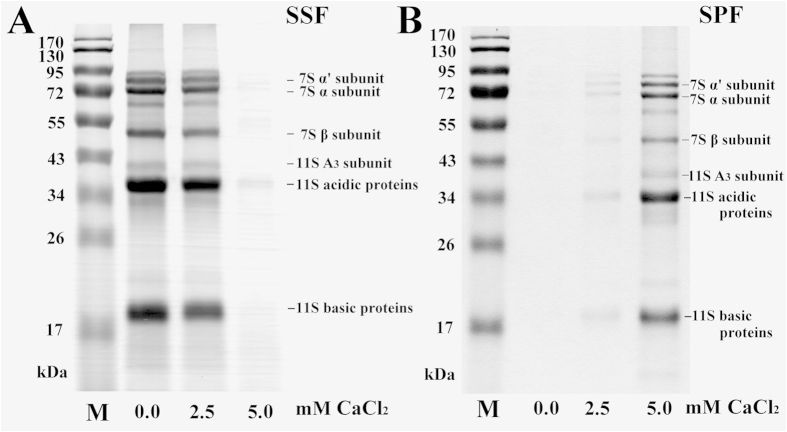
Changes in the SDS-PAGE profiles of soymilk with different amounts of calcium chloride (0, 2.5, or 5 mM) incubated at 30 °C for 1 h. (**A**) Soymilk supernatant fraction (SSF); (**B**) soymilk pellet fraction (SPF); M: protein marker.

As previously mentioned, the addition of 5 mM calcium chloride sufficiently coagulated the soymilk proteins into the SPF. Therefore, soymilk samples treated with varying amounts of calcium chloride (0, 2.5 or 5 mM) were analyzed by SDS-PAGE (Fig. 2). The intensity of the protein bands corresponding to the 7S α’, 7S α, 7S β, 11S A3, 11S acidic subunits and 11S basic proteins in the SSF significantly decreased following the addition of 5 mM calcium chloride (*P* < 0.05), the protein bands corresponding to the these proteins decreased to 1.8 ± 0.1, 0.9 ± 0.1, 1.1 ± 0.3, 1.6 ± 0.2, 2.1 ± 0.1 and 1.6 ± 0.1%, respectively (Fig. 2A). These proteins appeared in the SPF (Fig. 2B). The presence of 7S and 11S proteins in the SPF is evidence that calcium chloride binds to the 7S and 11S proteins and that they are both involved in soybean curd formation. The protein particles in soymilk contain a large amount of 7S β and 11S subunits and that the 11S subunit is formed by conjugation with the core of the 7S β subunit^17^. Teng *et al.*^18^ indicated that 10 mM and 20 mM calcium chloride can precipitate the maximum amounts of 11S and 7S, respectively. Our results suggest that 5 mM calcium chloride can precipitate soymilk proteins, including the 7S α’, 7S α, 7S β, 11S A3, 11S acidic subunits and 11S basic proteins.

### Identification of 7S and 11S proteins from 2-DE image of soymilk

**Figure 3:**
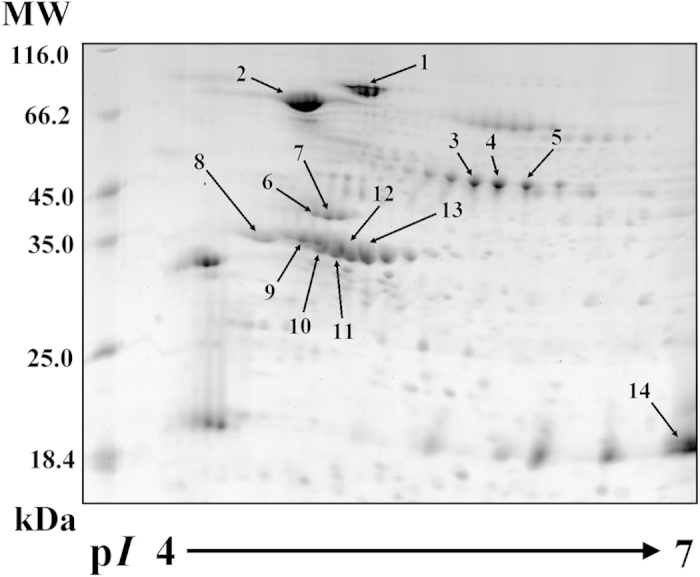
2-DE analysis of β-conglycinin and glycinin proteins in soymilk. Soymilk proteins were separated on a 12.5% SDS-PAGE gel using pH 4–7 IPG strips. MW: molecular weight; p*I*: isoelectric point. The arrows indicate the protein spots identified in this study and were grouped into isomers of the 7S α’ subunit (spot 1), 7S α subunit (spot 2), 7S β subunit (spots 3–5), 11S A3 subunit (spots 6–7), 11S A4 subunit (spot 8), 11S A2 subunit (spot 9), 11S A1b subunit (spots 10–11), 11S A1a subunit (spots 12–13), and 11S B1a subunit (spot 14).

A 2-DE image of the soymilk proteins is shown in Fig. 3. Fourteen protein spots selected from the 2-DE gel and digested with trypsin, and the resulting peptides were analyzed by MS. Fourteen of 7S and 11S proteins were identified, assigned individual numbers and cataloged according to their molecular weights (MW) and isoelectric points (p*I*) (Table 1). These proteins were grouped into isomers of the 7S α’ subunit (spot 1), 7S α subunit (spot 2), 7S β subunit (spots 3–5), 11S A3 subunit (spots 6–7), 11S A4 subunit (spot 8), 11S A2 subunit (spot 9), 11S A1b subunit (spots 10–11), 11S A1a subunit (spots 12–13), and 11S B1a subunit (spot 14). We found that several of the identified 7S and 11S proteins represented multiple observations of an individual soymilk protein on the 2-DE gel. The multiple spots on a 2-DE gel could be isoforms with different signals or target sequences, which would cause shifts in the p*I* and molecular weight^19^. The proteins could be post-translationally modified by the addition of side chains, phosphate, methyl groups, and other alterations that may affect the p*I* and molecular weight. Phosphorylation or glycosylation can also modify the molecular weight and/or p*I* of a protein^20^. Furthermore, the identified 7S (spots 1–5) and 11S (spots 6–14) proteins are storage proteins. The 7S is a trimeric protein composed of three subunits, α’, α and β, assembled as a result of hydrophobic forces and hydrogen bridges that may be combined in different ways^3^. Nielsen *et al.*^21^ indicated that 11S consists of five subunits: G1 (A1aB2), G2 (A2B1a), G3 (A1bB1b), G4 (A5A4B3) and G5 (A3B4). Several 11S subunits (spots 6–14), including A1a, A1b, A2, A3, A4 and B1a, were identified in our analyses. Based on physical properties, A1a, A1b, A2, A3, A4 and B1a are the subunits of G1, G3, G2, G5, G4 and G2.

### 2-DE analysis of the effects of calcium chloride on the 7S and 11S proteins

**Figure 4:**
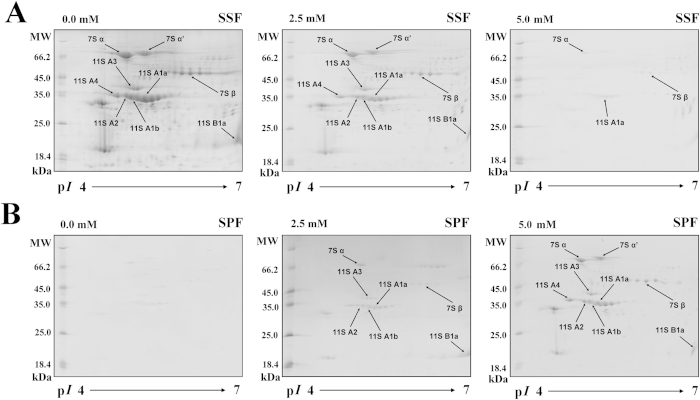
Changes in the two-dimensional polyacrylamide gel electrophoresis profiles of soymilk proteins after treatment with different amounts of calcium chloride (0, 2.5 or 5 mM) at 30 °C for 1 h. (**A**) soymilk supernatant fraction (SSF); (**B**) soymilk pellet fraction (SPF); M: protein marker; MW: molecular weight; p*I*: isoelectric point.

Soymilk samples treated with varying amounts of calcium chloride (0, 2.5 or 5 mM) were also analyzed by 2-DE gels (Fig. 4). When no calcium chloride treatment was employed, soymilk proteins were clearly observed in the SSF on the 2-DE gel, although no soymilk proteins were detected in the SPF. Some of the 7S and 11S proteins that were depleted in the SSF after 2.5 mM calcium chloride was added, including 7S α, 7S β, 11S A3, 11S A2, 11S A1b, 11S A1a, and 11S B1a, appeared in the SPF. Most of the soymilk proteins appeared in the SPF following the 5 mM calcium chloride treatment, and only a small amount of 7S α, 7S β and 11S A1a were observed in the SSF. Densitograms corresponding to the 2-DE images of the calcium chloride-treated SSF and SPF samples were also generated. As previously mentioned, most of the 7S subunits (spots 1–5) in the SSF were coagulated by the addition of 5 mM calcium chloride. The fold changes of the 7S α’ (spot 1), 7S α (spot 2), 7S β (spot 3), 7S β (spot 4) and 7S β (spot 5) subunits in the SSF were 0.04, 0.07, 0.03, 0.03 and 0.02, respectively. We observed that a portion of the 11S subunits (spots 6–14) in the SSF were also coagulated by the addition of 5 mM calcium chloride. The fold changes of 11S A3 (spot 6), 11S A3 (spot 7), 11S A4 (spot 8), 11S A2 (spot 9), 11S A1b (spot 10), 11S A1b (spot 11), 11S A1a (spot 12), 11S A1a (spot 13) and 11S B1a (spot 14) subunits in the SSF were 0.02, 0.01, 0.01, 0.02, 0.01, 0.03, 0.03, 0.01 and 0.12, respectively. These 7S and 11S proteins in spots 1–14 almost completely disappeared from the SSF and appeared in the SPF after the addition of 5 mM calcium chloride. Therefore, the coagulation of proteins, due to the addition of 5 mM calcium chloride, was demonstrated by the appearance of individual 7S and 11S proteins in the SPF.

### Isolation and identification of isoflavones from soymilk, 7S and 11S proteins

**Figure 5:**
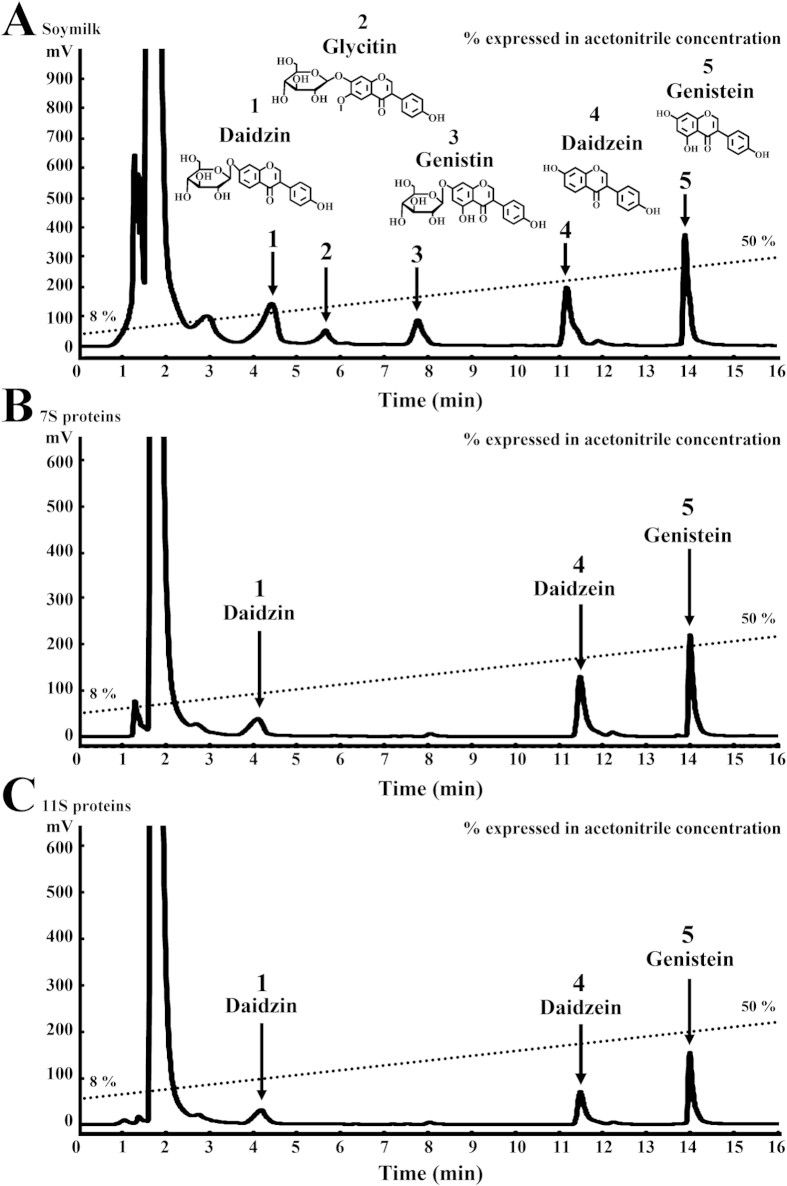
HPLC chromatograms of isoflavones extracted from the (**A**) soymilk, (**B**) 7S and (**C**) 11S proteins. Mobile phase: a gradient elution with H_2_O/acetonitrile (92:8, v/v,→50:50, v/v). Flow rate: 2 mL/min. Detection: UV 262 nm. Arrow: isoflavones. 1: daidzin. 2: glycitin. 3: genistin. 4: daidzein. 5: genistein.

The isoflavones extracted from soymilk and 7S and 11S proteins were analyzed by HPLC (Fig. 5). Isoflavones from soymilk were subjected to the HPLC (Fig. 5a). Five isoflavones (arrows, compounds 1–5) were isolated, which were collected at the elution times of 4–5, 5–6, 7–8, 11–12 and 14–15 min, respectively. The total yields of compounds 1–5 were 1.4, 1.0, 1.2, 1.1 and 1.3 mg, respectively. These purified isoflavones were analyzed using high-resolution ESI-TOF mass spectrometry, FT-IR and UV and were identified as daidzin, glycitin, genistin, daidzein and genistein, respectively. The quantification of these isoflavones by HPLC was performed, and commercial daidzin, glycitin, genistin, daidzein and genistein (LC Laboratories, Woburn, MA, USA) were used as the standards. The amount of each isoflavone in the soymilk (daidzin, glycitin, genistin, daidzein and genistein) were 20.2 ± 0.8, 3.3 ± 0.2, 6.9 ± 0.5, 15.7 ± 0.7 and 13.5 ± 0.1 μg/mL, respectively (Fig. 5A). The contents of daidzin, glycitin, genistin, daidzein and genistein in 95 °C heated soymilk were 0.50, 0.14, 0.59, 0.31 and 0.26 μmol/g dry basis, respectively^8^. The isoflavone content in soymilk could be affected by the ratio of water and soybean^22^. Among these isoflavones, daidzin, daidzein and genistein were isolated from 7S and 11S proteins, and the amount of each isoflavone in the 7S proteins were 1.4 ± 0.1, 2.5 ± 0.2 and 1.9 ± 0.1 μg/mg dry basis, respectively (Fig. 5B). The amount of each isoflavone in the 11S proteins (daidzin, daidzein and genistein) were 1.1 ± 0.1, 1.5 ± 0.1 and 1.5 ± 0.1 μg/mg dry basis (Fig. 5C). The contents of daidzin, daidzein and genistein in the 7S proteins were 0.25, 0.43 and 0.54 μmol/g dry basis, respectively^23^. Furthermore, the contents of daidzin, daidzein and genistein in the 11S proteins were 0.62, 0.39 and 0.41 μmol/g dry basis, respectively. The difference in the content of isoflavones between 7S and 11S might be due to structural differences in the proteins, causing a different affinity between proteins and isoflavones^24^.

The following sections will focus on the spectral data of the identified daidzin, glycitin, genistin, daidzein and genistein obtained from ESI-MS, FT-IR and UV.

### Daidzin (compound 1)

Compound 1 was a white powder (Fig. 5A), ESI-MS: *m/z* 417.1181 [M+H]^+^ (calculated for C_21_H_20_O_9_). IR *ν*_max_: 3411, 1634, 1515, 1446, 1249. UV (acetone: water: acetic acid = 70:29.5:0.5) *λ*_max_ nm: 232, 260. Compound 1 was identified as daidzin by direct comparison with commercial daidzin (LC Laboratories, Woburn, MA, USA). Based on the data described above and the verified reference^25^, the structure of compound 1 was elucidated as daidzin.

### Glycitin (compound 2)

Compound 2 was a white powder (Fig. 5A), ESI-MS: *m/z* 447.1286 [M+H]^+^ (calculated for C_22_H_22_O_10_). IR *ν*_max_: 3342, 1635, 1518, 1498, 1469, 1432, 1276, 1217, 1087, 1040. UV (acetone: water: acetic acid = 70:29.5:0.5) *λ*_max_ nm: 248, 220. Compound 2 was identified as glycitin by direct comparison with commercial glycitin (LC Laboratories, Woburn, MA, USA). Based on the data described above and the verified reference^26^, the structure of compound 2 was elucidated as glycitin.

### Genistin (compound 3)

Compound 3 was a white powder (Fig. 5A), ESI-MS: *m/z* 433.1130 [M+H]^+^ (calculated for C_21_H_20_O_10_). IR *ν*_max_: 3440, 1659, 1621, 1580, 1520. UV (acetone: water: acetic acid = 70:29.5:0.5) *λ*_max_ nm: 228, 260. Compound 3 was identified as genistin by direct comparison with commercial genistin (LC Laboratories, Woburn, MA, USA). Based on the data described above and the verified reference^25^, the structure of compound 3 was elucidated as genistin.

### Daidzein (compound 4)

Compound 4 was a white powder (Fig. 5A), ESI-MS: *m/z* 255.0651 [M+H]^+^ (calculated for C_15_H_10_O_4_). IR *ν*_max_: 3221, 1632, 1593, 1518. UV (acetone: water: acetic acid = 70:29.5:0.5) *λ*_max_ nm: 224, 248. Compound 4 was identified as daidzein by direct comparison with commercial daidzein (LC Laboratories, Woburn, MA, USA). Based on the data described above and the verified reference^6^, the structure of compound 4 was elucidated as daidzein.

### Genistein (compound 5)

Compound 5 was a white powder (Fig. 5A), ESI-MS: *m/z* 271.0601 [M+H]^+^ (calculated for C_15_H_10_O_5_). IR *ν*_max_: 3440, 3069, 2922, 1655, 1622, 1178, 810, 785. UV (acetone: water: acetic acid = 70:29.5:0.5) *λ*_max_ nm: 232, 260. Compound 5 was identified as genistein by direct comparison with commercial genistein (LC Laboratories, Woburn, MA, USA). Based on the data described above and the verified reference^27^, the structure of compound 5 was elucidated as genistein.

### HPLC analysis of the effects of calcium chloride on the isoflavones

**Figure 6:**
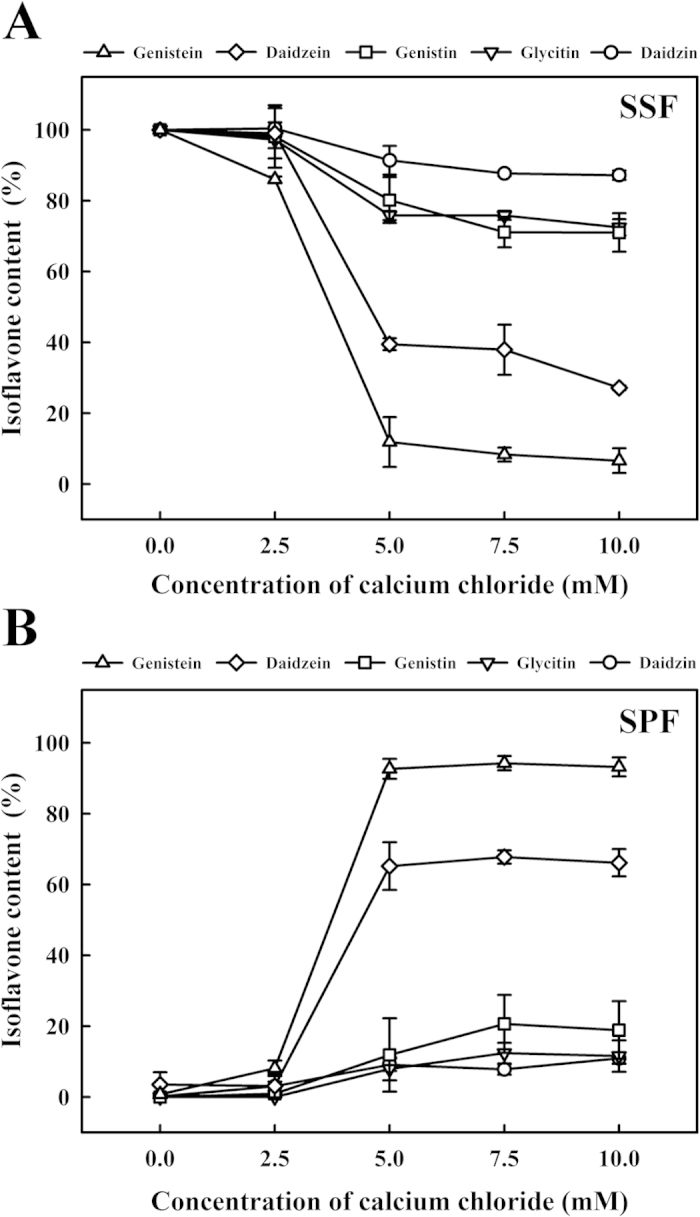
Changes in the isoflavones contents of soymilk with different amounts of calcium chloride (0, 2.5, 5, 7.5 or 10 mM) at 30 °C for 1 h. (**A**) soymilk supernatant fraction (SSF); (**B**) soymilk pellet fraction (SPF). Vertical bars represent standard deviations.

The isoflavone content in the SSF and SPF samples was determined by HPLC (Fig. 6). As shown in Fig. 6A, the amounts of daidzin, glycitin, genistin, daidzein and genistein in the SSF without calcium chloride were 19.2 ± 0.1, 3.4 ± 0.1, 7.3 ± 0.4, 16.4 ± 1.3 and 15.6 ±  0.6 μg/mL, respectively. Most of daidzein and genistein in the SSF significantly decreased with 5 mM calcium chloride (*P* < 0.05). However, only a portion of daidzin, gycitin and genistin in the SSF decreased with 5 mM calcium chloride. The amounts of daidzein, genistein, daidzin, gycitin and genistin in the SSF decreased to 39.4 ± 1.6, 11.8 ± 7.0, 91.4 ± 4.0, 75.8 ± 1.2 and 80.1 ± 6.4%, respectively, during that period. These isoflavones, including daidzein and genistein, and a portion of daidzin, gycitin and genistin also appeared in the SPF (Fig. 6B). This suggested that daidzein and genistein, and a portion of daidzin, gycitin and genistin, were coagulated from SSF into the SPF by calcium chloride.

**Figure 7:**
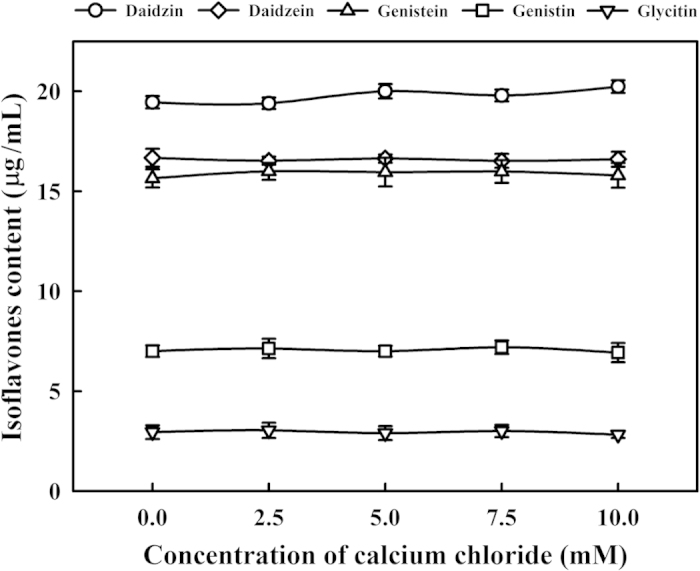
Changes in the isoflavones contents with different amounts of calcium chloride (0, 2.5, 5, 7.5 or 10 mM) at 30 °C for 1 h. Vertical bars represent standard deviations.

The effects of calcium chloride on the coagulation of isoflavones were also evaluated. The isoflavones extracted from soymilk were incubated with 0–10 mM of calcium chloride. However, no significant changes in the amounts of daidzin, daidzein, genistein, genistin and glycitin in supernatant were observed (Fig. 7). This result indicated that isoflavones were not immediately coagulated with calcium chloride. As previously mentioned, isoflavones including daidzin, daidzein and genistein were isolated from dried 7S and 11S proteins. The presence of daidzin, daidzein and genistein in the 7S and 11S proteins are evidence that daidzin, daidzein and genistein bind to the 7S and 11S proteins. The isoflavones may coprecipitate with proteins because of the surface hydrophobicity of the protein and its ability to interact with isoflavones^28^. Li and Hagerman^29^ suggested that the hydrophobic pocket between bovine serum albumin subdomains IIA and IIIA is the binding site for epigallocatechin-3-*O*-gallate. The interaction between flavanols and amino acids has also been reported. For example, glycine binds with the oxidized B-ring of catechin through the formation of Schiff bases^30^. Therefore, our results suggested that daidzein and genistein and a portion of daidzin, were bound with 7S and 11S proteins and then coprecipitated into the SPF by 5 mM calcium chloride.

In conclusion, we examined the effects of calcium chloride on the coagulation of β-conglycinin (7S), glycinin (11S), and isoflavones in soymilk. The results obtained from SDS-PAGE analysis, two-dimensional electrophoresis, and HPLC clearly demonstrate that the addition of 5 mM calcium chloride induced the coagulation of most of the 7S (α’, α and β), 11S acidic (A1a, A1b, A2, A3 and A4), and 11S basic (B1a) proteins was well as isoflavones, including daidzein and genistein. Moreover, we provide evidence that isoflavones (including daidzein and genistein) bonded to 7S and 11S to form 7S-isoflavone complex and 11S-isoflavone complex in soymilk. These complexes were then precipitated into SPF following the addition of 5 mM calcium chloride. Our findings provide important information related to the effects of calcium chloride on the coagulation of 7S, 11S, and isoflavones, and provide a valuable reference for future research into the production of tofu from soymilk.

## Materials and Methods

### Preparation of soymilk samples

Soybeans [*Glycine max* (L.) Merr., 100 g] were washed and soaked in distilled water at 25 °C for 12 h. Hydrated seeds were drained and ground into homogenates in 1 L of distilled water. The raw soymilk was filtered through a cotton filter and heated in a 90 °C water bath for 1 h. The soymilk was collected and stored at 4 °C. To investigate the effects of calcium chloride on the coagulation of soymilk proteins, soymilk was centrifuged at 12,000 × g for 20 min at 4 °C using a centrifugal separator to remove lipids. Varying concentrations of calcium chloride (0, 2.5, 5, 7.5 or 10 mM) were added to 1 mL of soymilk, followed by incubation at 30 °C for 1 h. Following incubation, the soymilk samples were fractionated into the soymilk supernatant fraction (SSF) and the soymilk pellet fraction (SPF) by centrifugation for 20 min (5,000 × g). The SSF samples (1 mL) were collected, and the SPF samples were re-dissolved in an equal volume (1 mL) of a lysis solution containing 7 M urea, 2 M thiourea, and 4% 3-[(3-cholamidopropyl)-dimethylammonio]-1-propanesulfonate prior to use.

### Preparation of 7S and 11S proteins

The preparation of 7S and 11S proteins from soymilk was according to the method of Nagano *et al.*^31^ with some modifications. Briefly, 0.1% sodium bisulfate was added to soymilk (25 mL), and the pH was adjusted to 6.4 with 0.2 M HCl and then incubated at 4 °C for 12 h. After 20 min centrifugation at 12,000 × g at 4 °C, the pellet (11S protein) was collected. NaCl (0.25 M) was added to the supernatant, and the pH was adjusted to 5.0 with 0.2 M HCl and incubated at 4 °C for 1 h. After 20 min centrifugation at 12,000 × g at 4 °C, the supernatant was collected and adjusted to pH 4.8 with 0.2 M HCl. After 20 min centrifugation at 12,000 × g at 4 °C, the pellet (7S protein) was collected. The protein concentrations of samples were determined using a Bio-Rad protein assay kit (Bio-Rad, Hercules, CA, USA). Bio-Rad protein assay dye was diluted with 4 volumes of water and mixed with standards or soymilk samples. The absorbance at 595 nm was measured using a UV spectrophotometer (U-1900; Hitachi High-Technologies Corporation, Minato-ku, Japan), and bovine serum albumin (Sigma Chemical Co., St. Louis, MO, USA) was used as the standard.

### Preparation of isoflavone samples

Soymilk, SSF, SPF, 7S and 11S proteins samples were lyophilized into powder and the isoflavones were extracted from these samples according to the method of Xu and Chang^32^. Briefly, each sample was extracted with 10 mL solvent (acetone: water: acetic acid = 70:29.5:0.5, v/v/v) for 3 h at 30 °C. After 10 min centrifugation at 10,000 × g at 4 °C, the supernatants (isoflavones) were subjected to HPLC analysis. The effects of calcium chloride on the coagulation of isoflavones were also evaluated. The isoflavones (60 μg/mL) extracted from soymilk were incubated with varying concentrations of calcium chloride (0, 2.5, 5, 7.5 or 10 mM) for at 30 °C for 1 h. After 20 min centrifugation at 12,000 × g at 4 °C, the supernatants were subjected to HPLC analysis.

### Sodium dodecyl sulfate polyacrylamide gel electrophoresis (SDS-PAGE)

SDS-PAGE analysis of the SSF and SPF samples was performed with a 12.5% separating gel and a 5% stacking gel. An aliquot of 0.1 mL of each sample was mixed with 0.9 mL of buffer (2% SDS, 5% β-mercaptoethanol, 10% glycerol, 0.02% bromophenol blue and 70 mM Tris-HCl, pH 6.8) and heated at 95 °C for 5 min. The samples (6 μL) and a protein ladder were loaded into separate wells. Following electrophoresis, the gels were stained with Coomassie brilliant blue R-250. The stained gels were digitized using an Epson perfection 1270 image scanner (Epson America Inc., Long Beach, CA, USA) and analyzed using Quantity One 1-D analysis software (version 4.6.3, Bio-Rad laboratories, Berkeley, CA, USA) and Gel-Pro Analyzer software (version 4.0, Media Cybernetics, Inc., Bethesda, MD, USA). Changes in electrophoretic profiles were used to evaluate the protein coagulation induced by calcium chloride.

### Two-dimensional electrophoresis (2-DE)

Soymilk samples were analyzed using 2-DE. For the first separation, samples were immobilized and loaded onto pH gradient gel (IPG) strips (pH 4–7, 18 cm, GE Healthcare), which had been rehydrated for 12 h in a solution containing 7 M urea, 2 M thiourea, 4% 3-[(3-cholamidopropyl)-dimethylammonio]-1-propanesulfonate, 4 mM Tris-base, 2% IPG ampholyte, 65 mM 1,4-dithioerythritol (DTE) and 0.0002% bromophenol blue prior to use. Isoelectric focusing of the strips was performed using the IPGphor 3 IEF system (GE Healthcare, Waukesha, WI, USA) at 20 °C at 6000 V, for a total of 60 kVh. The strips were equilibrated for 15 min in an equilibration solution (50 mM Tris-HCl, pH 8.8, 6 M urea, 2% SDS, 30% glycerol and 2% DTE) and added to the top of a vertical 12.5% SDS-PAGE gel with 0.5% agarose. The second electrophoresis step was performed using the Protean II xi Cell system (Bio-Rad, Hercules, CA, USA) at 10 mA per gel for 1 h and 45 mA per gel for 5 h until the bromophenol blue reached the bottom of the gel. After electrophoresis, the gels were immersed in 10% methanol and 7% acetic acid for 30 min and stained in 350 mL of Sypro^®^ Ruby protein gel stain solution overnight. The developed gels were digitally scanned as 2-DE images with a Typhoon 9200 imaging system (Amersham Pharmacia Biotech, Uppsala, Sweden) and analyzed using the PDQuest software package (version 7.3, Bio-Rad, Hercules, CA, USA).

### Protein digestion and identification

Fourteen protein spots on the 2-DE were excised and destained in a solution containing 250 μL acetonitrile/50 mM ammonium bicarbonate (1:1). The gels were dried using a centrifugal vacuum concentrator. The cysteine residues in the samples were reduced and alkylated using DTE and iodoacetamide, respectively. The gels were rehydrated in a trypsin solution (12.5 ng/mL) for tryptic digestion and incubated at 37 °C for 16 h. Peptide fragments were extracted with an equal volume of 100% acetonitrile and 2% trifluoroacetic acid, and sonicated in a water bath for 10 min. The extracted peptides were concentrated in a vacuum centrifuge. For matrix-assisted laser desorption/ionization-mass spectrometry (MALDI-MS) analysis, the extracted peptides were mixed 1:1 with a matrix solution (5 mg/mL α-cyano-4-hydroxycinnamic acid in 50% acetonitrile, 0.1% trifluoroacetic acid and 2% ammonium citrate) and spotted onto a 96-well format MALDI sample stage. Data were directly obtained using a quadrupole time-of-flight (Q-TOF) Ultima MALDI instrument (MALDI^TM^; Micromass, UK). Peptide mass fingerprint data from MALDI-Q-TOF analyses were used to identify protein candidates in the Swiss-Prot protein database using the Mascot (http://www.matrixscience.com) search program to identify proteins. The search parameters allowed for methionine oxidation, cysteine carbamidomethylation, one missed cleavage site and a peptide mass tolerance of 0.25 Da. The product ion spectra generated by Q-TOF MS/MS were compared to the Swiss-Prot protein database, and an exact match was found using the Mascot search program.

### HPLC analysis and identification of the isoflavones

The isoflavone samples were subjected to column chromatography on the HPLC according to the method of Hsieh, Kao, and Chen^7^. The HPLC system is composed of a pump (PU-2089, JASCO), a detector (UV-2070, JASCO) and a C18 packed column (Vydac 218TP54, 4.6 mm × 250 mm, 5 μm Spherical, Grace Vydac, Hesperia, CA, USA). The mobile phase was solvent A (100% acetonitrile) and solvent B (ultrapure water). Elution conditions were as follows: 8–50% of A to B for 0–16 min (linear gradient) at a flow rate of 2 mL/min. The effluent was monitored through an optical absorption study carried out at 262 nm. The isoflavones were then collected, and the chemical structures were identified using ultraviolet spectra (UV, Hitachi, U-1900), Fourier transform infrared spectrum (FT-IR, Avatar 320, Pittsfield, MA. USA), a polarity meter (Perkin Elmer, 341), and high-resolution ESI-TOF mass spectrometry (ESI-MS, BioTOF III; Bruker Daltonics, Inc. Billerica, MA, USA).

### Statistical analysis

Data were expressed as the means ± standard deviations. The data were analyzed using Statistical Package for the Social Sciences software (SPSS for Windows, version 10.0.7C, SPSS Inc., Chicago, IL, USA). The statistical significance between the treatments was determined by one-way ANOVA followed by Duncan’s multiple range test. Three determinations for each treatment were performed, and the significance level was set at *P* < 0.05.

## Additional Information

**How to cite this article**: Hsiao, Y.-H. *et al.* Coagulation of β-conglycinin, glycinin and isoflavones induced by calcium chloride in soymilk. *Sci. Rep.* **5**, 13018; doi: 10.1038/srep13018 (2015).

## Figures and Tables

**Table 1:** β-conglycinin (7S) and glycinin (11S) proteins identified and cataloged from 2-DE^a^.

Spot No.^b^	Entry name	Protein name	MW^c^ (Da)/p*I*^d^		MS^e^ method	Mowse score	% Coverage	Species
			Apparent	Theoretical				
1	7S, α’ subunit	GLCAP_SOYBN	85000/5.3	72203/5.51	MS/MS	127	11	*Glycine max*
2	7S, α subunit	GLCA_SOYBN	72000/4.8	70250/5.07	MS/MS	174	8	*Glycine max*
3	7S, β subunit	GLCB_SOYBN	55000/5.9	50521/5.88	MS/MS	88	11	*Glycine max*
4	7S, β subunit	GLCB_SOYBN	55000/6.1	50521/5.88	MS/MS	72	6	*Glycine max*
5	7S, β subunit	GLCB_SOYBN	55000/6.3	50521/5.88	MS/MS	110	9	*Glycine max*
6	11S, A3 subunit	GLYG5_SOYBN	42000/5.0	57921/5.60	MS/MS	86	8	*Glycine max*
7	11S, A3 subunit	GLYG5_SOYBN	42000/5.1	57921/5.60	MS/MS	100	7	*Glycine max*
8	11S, A4 subunit	GLYG4_SOYBN	38000/4.8	63548/5.29	MS/MS	40	6	*Glycine max*
9	11S, A2 subunit	GLYG2_SOYBN	37000/5.0	54357/5.46	MS/MS	31	2	*Glycine max*
10	11S, A1b subunit	GLYG3_SOYBN	37000/5.1	54208/5.73	MS/MS	38	3	*Glycine max*
11	11S, A1b subunit	GLYG3_SOYBN	37000/5.2	54208/5.73	MS/MS	36	3	*Glycine max*
12	11S, A1a subunit	GLYG1_SOYBN	37000/5.3	55672/5.89	MS/MS	51	7	*Glycine max*
13	11S, A1a subunit	GLYG1_SOYBN	37000/5.4	55672/5.89	MS/MS	40	7	*Glycine max*
14	11S, B1a subunit	GLYG2_SOYBN	23000/6.9	54357/5.46	MS/MS	82	3	*Glycine max*

^a^2-DE = two-dimensional gel electrophoresis.

^b^Spot No. = spot number, corresponding to Fig. 3.

^c^MW = molecular weight.

^d^p*I* = isoelectric point.

^e^MS = mass spectrometry.
